# Engineering base-excised aptamers for highly specific recognition of adenosine†

**DOI:** 10.1039/d0sc00086h

**Published:** 2020-02-10

**Authors:** Yuqing Li, Biwu Liu, Zhicheng Huang, Juewen Liu

**Affiliations:** Department of Chemistry, Waterloo Institute for Nanotechnology, University of Waterloo Waterloo Ontario N2L 3G1 Canada liujw@uwaterloo.ca

## Abstract

The DNA aptamer for adenosine and ATP has been used as a model system for developing analytical biosensors. For practical reasons, it is important to distinguish adenosine from ATP, although this has yet to be achieved despite extensive efforts made on selection of new aptamers. We herein report a strategy of excising an adenine nucleotide from the backbone of a one-site adenosine aptamer, and the adenine-excised aptamer allowed highly specific binding of adenosine. Cognate analytes including AMP, ATP, guanosine, cytidine, uridine, and theophylline all failed to bind to the engineered aptamer according to the SYBR Green I (SGI) fluorescence spectroscopy and isothermal titration calorimetry (ITC) results. Our A-excised aptamer has two binding sites: the original aptamer binding site in the loop and the newly created one due to base excision from the DNA backbone. ITC demonstrated that the A-excised aptamer strand can bind to two adenosine molecules, with a *K*_d_ of 14.8 ± 2.1 μM at 10 °C and entropy-driven binding. Since the wild-type aptamer cannot discriminate adenosine from AMP and ATP, we attributed this improved specificity to the excised site. Further study showed that these two sites worked cooperatively. Finally, the A-excised aptamer was tested in diluted fetal bovine serum and showed a limit of detection of 46.7 μM adenosine. This work provides a facile, cost-effective, and non-SELEX method to engineer existing aptamers for new features and better applications.

## Abstract 2

The DNA aptamer for adenosine and ATP has been used as a model system for developing analytical biosensors.

## Graphical abstract

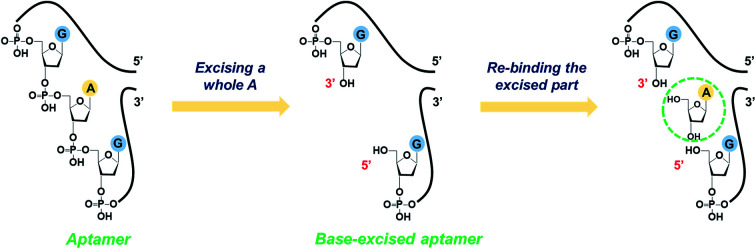

## Introduction

Aptamers are single-strand oligonucleotides that can specifically bind to target molecules.^1–3^ Compared to antibodies, DNA aptamers are much more stable and cost-effective. Some aptamers are naturally found in riboswitches, which can regulate gene expression and interact with metabolites, such as adenine, flavin mononucleotides, and glycine.^4^ Most aptamers used for designing analytical biosensors are generated through a strategy called SELEX (systematic evolution of ligands by exponential enrichment).^5,6^ SELEX has been applied to various targets ranging from metal ions,^7,8^ small molecules,^9,10^ peptides,^11^ proteins,^12^ to whole cells.^13^ Due to these advantages, aptamers are widely used in biosensing,^14,15^ cell engineering,^16^ and therapeutics.^17^

The DNA aptamer for ATP or adenosine is one of the most important model aptamers. It was first reported by Huizenga and Szostak in 1995, having excellent selectivity against the other nucleosides or nucleotides.^9^ With more than 20 years of research, its structure,^18^ highly conserved nucleotides,^19,20^ modification,^3^ and single-site binding mutants^21^ have been revealed. Nevertheless, this aptamer is limited by its inability to distinguish the substituents on the 5′ position of adenosine (*e.g.* cannot distinguish adenosine from AMP, cAMP, or ATP). Since adenosine regulates different cellular processes, and affects the immune, nervous, respiratory, circulatory and urinary systems, abnormal levels of adenosine indicate potential problems in the heart and brain.^22,23^ Therefore, it is important to differentiate adenosine from AMP and ATP.

To improve the aptamer specificity, much effort has been made. For example, Koizumi and Breaker selected a new aptamer that can differentiate cAMP from ATP, 5′-AMP and 3′-AMP.^24^ Through extensive negative selections, the Szostak group isolated an RNA aptamer for ATP, which specifically targeted the triphosphate moiety.^25^ Olsen *et al.* obtained an aptamer that can monitor the oscillating concentration of ATP with high resolution, even if the total concentration of adenine nucleotides ([ATP] + [ADP] + [AMP]) was stable.^26^ Nutiu and Li re-discovered the ATP DNA aptamer using a new SELEX method,^19^ and Ellington *et al.* selected a fluorescein-labeled ATP RNA aptamer.^27^ Most recently, Zheng *et al.* reported an ATP aptamer that can be used in cytometric bead assays.^28^ Although these SELEX strategies are powerful for guiding the selectivity, still no aptamers are available for highly selective binding of adenosine.

Since DNA oligonucleotides are chemically synthesized, they allow efficient mutation and modification studies.^29–31^ Recently, it was reported that after omitting a base on a DNA duplex or G-quadruplex, the scaffold allowed free adenosine or guanosine to re-fit into the vacant site. This was achieved either by introducing an abasic-site in the duplex,^32–34^ or by omitting a whole guanine-nucleotide in a G-quadruplex.^35–37^ These DNA complexes can reach a μM *K*_d_, and behave like traditional aptamers. However, they often have a low specificity. Not only the cognate analytes can fit into the vacancy,^36^ pseudo-base pairing may also induce binding.^32,38^ This may be attributed to their recognition largely relying on secondary structural discrimination (simple base pairing).

To achieve more specific recognition, herein we reported an interesting finding of using an aptamer as the scaffold to exclusively recognize adenosine. By deleting a nucleotide in the adenosine aptamer, a new strand breaking point was created, and we called it a base-excised aptamer. Compared with scaffolds consisting of a duplex and a G-quadruplex, the base-excised aptamer has more sophisticated 3D structures that may allow more intermolecular forces to take place and enable exclusive adenosine recognition. Other nucleosides including cytidine, guanosine, uridine, and cognate analytes like AMP, ATP and theophylline all failed to bind. This work provides insights into engineering existing aptamers without the need of new SELEX experiments.

## Materials and methods

### Chemicals

All the DNA samples were purchased from Eurofins (Huntsville, AL). The DNA sequences are listed in ESI Table S1.† SYBR Green I (SGI), adenosine monophosphate (AMP), adenosine triphosphate (ATP), theophylline and fetal bovine serum (FBS) were purchased from Sigma-Aldrich. Guanosine, adenosine, cytidine, uridine, 4-(2-hydroxyethyl)piperazine-1-ethanesulfonate (HEPES), sodium chloride and magnesium chloride were from Mandel Scientific (Guelph, Ontario, Canada). Milli-Q water was used to prepare all of the buffers and solutions.

### SGI-based binding assays

An aptamer (50 nM) was first incubated with 2 mM target molecules, including adenosine, AMP, ATP, guanosine, cytidine or uridine, for 5 min in buffer A (20 mM HEPES, pH 7.6, containing 100 mM NaCl and 5 mM MgCl_2_). The aptamer incubated with the buffer (without target) was used as control. Then, 50 nM SGI (final concentration) was added and the fluorescence spectra were collected (excitation at 485 nm, emission at 535 nm). The fluorescence data were analyzed based on (*F*_0_ − *F*)/*F*_0_, in which *F*_0_ and *F* stand for the fluorescence without and with the target molecule, respectively. Similarly, a duplex DNA (50 nM) was first incubated with 2 mM cytidine or adenosine for 5 min in buffer A, and then 12.5 nM SGI was added. The same sample without adding adenosine or cytidine was used as control.

### Isothermal titration calorimetry (ITC)

ITC was performed using a VP-ITC microcalorimeter instrument (MicroCal). All samples were ultrasonicated for 5 min (degassing) prior to applying them for ITC. A 20 μM one-site aptamer, Res-A10-Right cut, and the A10-excised aptamer (in buffer A) were loaded in a 1.45 mL cell at 10 °C, respectively. 1 mM adenosine or AMP in the same buffer was loaded in a 280 μL syringe. After the first injection of 2 μL, the syringe injected 10 μL of the target into the cell each time. Through measuring the heat changes and fitting the titration curves to a one-site binding model, thermodynamic data including the association constant (*K*_a_), enthalpy change (Δ*H*), entropy change (Δ*S*), free energy change (Δ*G*), and binding stoichiometry (*N*) were obtained. The molar ratio was calculated from the ITC data based on the ligand/aptamer concentrations.

### Detection in diluted serum

1% fetal bovine serum (FBS) was used for this study. First, 1% FBS was prepared by diluting the serum sample in 20 mM HEPES (pH 7.6, containing 100 mM NaCl, 5 mM MgCl_2_). Then 50 nM DNA was incubated with or without 2 mM adenosine in 1% FBS, and stained by 50 nM SGI. To evaluate its specificity, the DNA was also incubated with 2 mM AMP in the 1% FBS. At last, different concentrations of adenosine (0.28 to 2 mM) were titrated to calculate the *K*_d_ value.

## Results and discussion

### Abasic, base-spliced and base-excised DNA

**Fig. 1:**
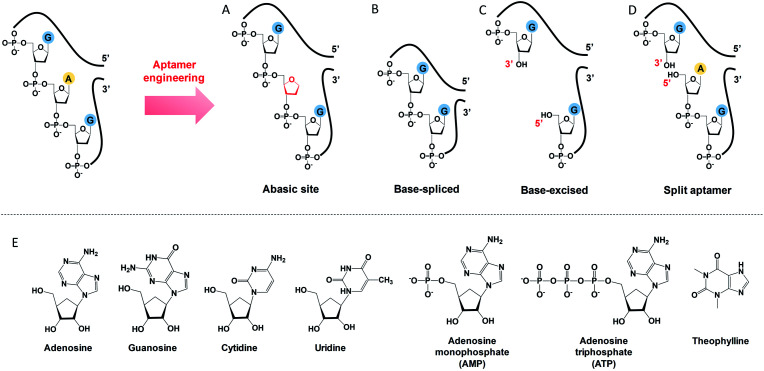
A scheme showing the differences between (A) abasic site, (B) base-spliced, (C) base-excised and (D) split aptamers. Note that the 5′ terminus of the purchased DNA was –OH (chemically synthesised), rather than a phosphate. (E) Structures of the analytes used: adenosine, guanosine, cytidine, uridine, AMP, ATP and theophylline.

To engineer a vacancy-bearing DNA for specifically refilling the vacancy, two strategies have already been reported. One is to create an abasic site by breaking the *N*-glycosylic bond (Fig. 1A),^39^ where the number of phosphodiester bonds remains the same but one of the bases is removed. The other method is to fully remove an entire nucleotide, and join the cleaved strands neatly at the end, which we call the base-spliced strategy (Fig. 1B).^35,36^ Although rebinding of the deleted parts has been demonstrated in both methods, the specificity remained poor. For example, the abasic-site-contained DNA duplex can bind both adenosine and theophylline based on cytosine recognition,^32,40^ and the vacancy-bearing G-quadruplex can accept guanosine, GMP, GDP and GTP with similar affinities.^36^

In our work here, a new method of DNA engineering is described in Fig. 1C, in which a whole nucleotide is excised, and a break is created (a new 3′ end and a new 5′ end). This is different from the simple splitting (Fig. 1D), which creates a break but does not remove any base. Besides, the goal of split aptamers is not to detect the removed nucleotide.^41–43^ To demonstrate a better specificity of our base-excised aptamer for adenosine, not only the analogues focused on the base part (*i.e.* guanosine, cytidine, uridine and theophylline), but also the substituents on the 5′ position like AMP and ATP were carefully tested (Fig. 1E).

### Design of the adenine-excised adenosine aptamer

**Fig. 2:**
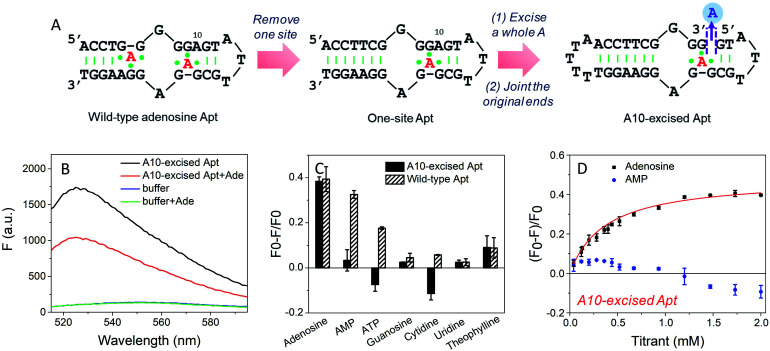
(A) The secondary structure of the wild-type adenosine aptamer, the engineered one-site aptamer and the A10-excised aptamer. (B) Fluorescence spectra of 50 nM A10-excised aptamer mixed with or without 2 mM adenosine in buffer A (20 mM HEPES, 100 mM NaCl, and 5 mM MgCl_2_). 50 nM SGI (final concentration) was used. (C) Comparison of the specificity of the A10-excised aptamer with the wild-type aptamer. 50 nM DNA mixed with 2 mM testing molecules, including adenosine, AMP, ATP, guanosine, cytidine and uridine in buffer A with 50 nM SGI. (D) Titrating adenosine or AMP (40 μM to 2 mM) with 50 nM A10-excised aptamer in buffer A. The titration curve was fitted by the equation: *F* = *F*_0_ − *a*[adenosine]/(*K*_d_ + [adenosine]).

To study the molecular recognition in base-excised DNA, the adenosine aptamer was chosen as a scaffold. The secondary structure of the wild-type aptamer is shown in Fig. 2A as revealed by Lin and Patel.^18^ The red A stands for an adenine-containing ligand, such as adenosine, AMP, ADP and ATP, all of which can be accommodated in the aptamer binding pockets. The binding pockets contain G·A mismatches, and around them are some stacked bases, including the flanked G·G mismatch in one direction and the G·A mismatch in another direction. Therefore, hydrogen bonding and base stacking are main contributors for target recognition.^20^

We decided to first excise the A10 adenine within the binding pockets because this position is close to the target binding site. Since the original aptamer can bind two adenosine molecules and it may complicate data analysis, we used the one-site aptamer design by sealing the base pairs on the left side of the aptamer (Fig. 2A, middle sequence). This one-site aptamer has a similar binding affinity and specificity for adenosine.^21^ After excising a whole A10, two strands were generated. To keep the excised-aptamer in one strand, its original 3′ and 5′ ends were joined together, and a new aptamer was generated from the 5′ end near the excised site. The final sequence was named the A10-excised aptamer (Fig. 2A, right one).

### Adenosine specifically binds to the A10-excised aptamer

To study the binding performance of the A10-excised aptamer, SYBR Green I (SGI) was used for label-free binding assays.^44–48^ First, a 50 nM A10-excised aptamer was incubated with 2 mM adenosine (Fig. 2B). Compared with the DNA without adenosine, the fluorescence decreased around 40% at 485 nm (excitation at 535 nm), indicating the binding of adenosine. This SGI fluorescence decreasing trend was consistent with previously reported studies, which was attributable to displacement of SGI by adenosine in the binding pockets.^46–48^ We then, respectively, incubated AMP, ATP, guanosine, cytidine, uridine and theophylline (2 mM each) with the DNA (Fig. 2C, black bars). Interestingly, they all failed to decrease the fluorescence signal, suggesting a lack of binding. For comparison, we also tested the wild-type aptamer with the sequence shown in Fig. 2A. A descending binding trend was observed with adenosine, AMP and ATP, whereas negligible fluorescence was found for the other three nucleosides and theophylline. Therefore, using this base excised aptamer, the specificity for adenosine drastically improved.

To quantitatively measure binding, we further titrated up to 2 mM adenosine and AMP into the A10-excised aptamer (Fig. 2D). Adenosine showed a *K*_d_ of 0.37 mM, while AMP failed to bind at any of these concentrations. Therefore, our A10-excised aptamer not only distinguished adenosine from C, G and T, but also distinguished the phosphate part showing no binding to even AMP. This adenosine/AMP distinction has been a main problem of the original aptamer, which has hindered data interpretation, since the cellular adenosine pool contains various AMP, ADP, AMP and even cAMP.^49^ It could be that the phosphate containing nucleotides are negatively charged and thus are more repelled by the charges on the phosphate backbone of the DNA, while the charge neutral adenosine is easier to bind.

Since the original aptamer pocket can accommodate all the adenosine derivatives, we attributed this excellent specificity to the excised site, which may only bind adenosine, but not AMP. The aptamer pocket can only form when this A10 site was filled with adenosine. To test this, we designed a few control experiments. We incubated the A10-excised aptamer with 0.5 mM adenosine (half saturation according to Fig. 2D), and then titrated AMP (Fig. S1†). The fluorescence increased, suggesting that AMP competed with adenosine and disrupted the binding complex (orange curve). This suggests that both sites had to bind adenosine and one AMP and one adenosine were not optimal. Our A10-excised aptamer bears two adenosine binding pockets, with one in the backbone and the other in the loop. Based on the fluorescence data, the improved specificity for adenosine was due to the excision of the A from the backbone.

### Splitting at the A10-site (no base excised)

**Fig. 3:**
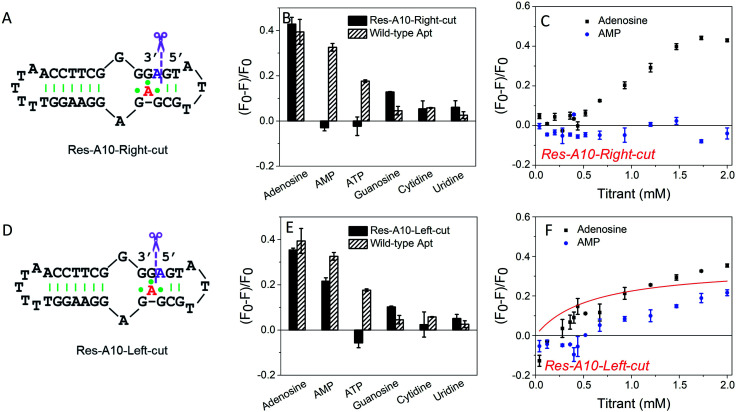
(A) Secondary structure of the Res-A10-Right-cut. (B) Comparison of specificity of the Res-A10-Right-cut and the wild-type aptamer. 50 nM DNA mixed with 2 mM testing molecules in buffer A (20 mM HEPES, 100 mM NaCl, and 5 mM MgCl_2_) with 50 nM SGI. (C) Titrating adenosine or AMP into 50 nM Res-A10-Right-cut in buffer A. (D) Secondary structure of the Res-A10-Left-cut. (E) Comparison of specificity of the Res-A10-Left-cut and wild-type aptamer. (F) Titrating adenosine or AMP (40 μM to 2 mM) into the Res-A10-Left-cut.

To further understand the effect of the above design, we then split the aptamer at the A10-site (at the right and left sides of A10, respectively), but without any nucleotide excised. We first cut the right-side phosphodiester bond (Fig. 3A). Interestingly, it showed similar binding specificity to the A10-excised aptamer, in which only the adenosine induced ∼42% fluorescence decrease, whereas the AMP, ATP, cytidine and uridine were all failed to achieve a fluorescence signal change (Fig. 3B). The titration data further confirmed the binding of adenosine, while no AMP binding was observed (Fig. 3C). Half saturation was achieved at 0.94 mM adenosine, and thus this splitting modification near the aptamer binding pocket also decreased the binding affinity.

In contrast, when cutting the phosphodiester bond on the left side (Fig. 3D), the binding profiles were different (Fig. 3E). AMP also showed binding, and its fluorescence drop was about two thirds of that of adenosine. From the titration in Fig. 3F, the Res-A10-Left-cut binds adenosine with a *K*_d_ of 0.57 mM. These experiments indicated that the right-side and the left-side phosphodiester bonds around the A10-site played different roles in helping the loop pockets bind AMP. Breaking the right-side one might change the aptamer local folding, resulting in differentiated binding preference for adenosine and AMP. For both split aptamers, the observed binding was from the original aptamer binding pocket. Therefore, it is not surprising that AMP can also bind in some cases.

### Studying binding thermodynamics using ITC

**Fig. 4:**
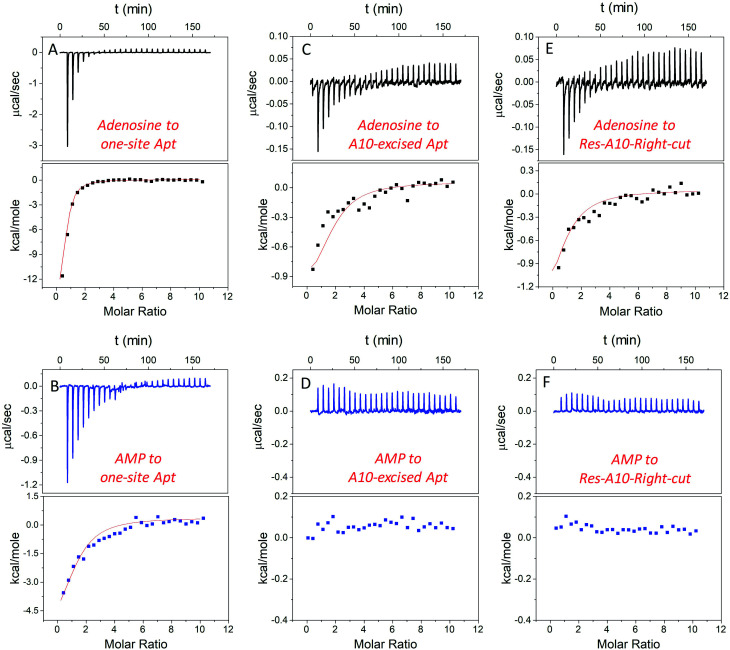
ITC traces and integrated heat for 20 μM one-site aptamer titrated by (A) 1 mM adenosine and (B) AMP in buffer A (20 mM HEPES, pH 7.6, 100 mM NaCl, and 5 mM MgCl_2_); 20 μM A10-excised aptamer titrated by (C) 1 mM adenosine and (D) AMP in buffer A; and 20 μM Res-A10 aptamer titrated by (E) 1 mM adenosine and (F) AMP in buffer A. All the titrations were carried out at 10 °C to promote aptamer binding, and each titration was repeated at least twice. The error for fitting the data was directly obtained from the software in the ITC.

In addition to studying the aptamer binding using fluorescence spectroscopy, we also employed isothermal titration calorimetry (ITC). ITC can quantitatively measure the heat changes during the binding process. This can provide rich thermodynamic information, including the association constant (*K*_a_), enthalpy changes (Δ*H*), entropy changes (Δ*S*), free energy changes (Δ*G*), and binding stoichiometry (*N*).^21,50–52^Fig. 4A and B display typical ITC traces of titrating adenosine and AMP into the one-site aptamer, consisting of the upper part of downward spikes and the lower part of the fitted integrated heat. The one-site aptamer was firstly titrated to confirm its similar binding abilities to the wild-type aptamer, which cannot distinguish adenosine from AMP. The *K*_d_ of the wild-type aptamer binding to adenosine was reported to be around 6 μM (room temperature) in the original paper,^9^ and here the *K*_d_ for one-site aptamer binding to adenosine was measured to be 2.8 ± 0.1 μM (10 degree for all titrations, Table 1). The *K*_d_ for the one-site aptamer binding to AMP was 13.5 ± 1.6 μM. Therefore, the aptamer binds adenosine slightly tighter than AMP.

**Table 1:** Thermodynamic data of aptamers binding to adenosine and AMP (at 10 °C)

Ligands	Aptamers	*N*	*K*_a_ (×10^4^ M^−1^)	*K*_d_ (μM)	Δ*G* (kcal mol^−1^)	Δ*H* (kcal mol^−1^)	Δ*S* (cal K^−1^ mol^−1^)
Adenosine	One-site Apt	0.8 ± 0.1	35.7 ± 1.4	2.8 ± 0.1	−6.8 ± 1.9	−13.5 ± 1.9	−22.4
	Res-A10-Right-cut	0.9 ± 0.2	4.8 ± 0.8	21.0 ± 3.8	−5.6 ± 0.3	−1.1 ± 0.3	13.5
	A10-excised Apt	1.8 ± 0.3	6.8 ± 0.9	14.8 ± 2.1	−6.5 ± 0.3	−1.0 ± 0.3	18.7
AMP	One-site Apt	1.3 ± 0.3	7.39 ± 0.9	13.5 ± 1.6	−6.4 ± 0.8	−4.5 ± 0.8	6.5
	Res-A10-Right-cut		—a				
	A10-excised Apt		—a				

a Binding was extremely weak and cannot be obtained by ITC.

We then titrated adenosine and AMP to the A10-excised aptamer and the Res-A10-Right-cut, respectively (Fig. 4C–F). Consistent with the fluorescence data demonstrated above, they both only bound to adenosine, whereas they failed to accept AMP. The *K*_d_ of the A10-excised aptamer binding to adenosine was 14.8 ± 2.1 μM, and that to the Res-A10-Right-cut was 21.0 ± 3.8 μM. The *K*_d_ of them binding to AMP cannot be obtained from the ITC due to undetectable heat changes. It is interesting to note that the *K*_d_ values obtained from ITC indicated tighter binding than those from SGI fluorescence spectroscopy. It could be that SGI affected the binding. Nevertheless, the conclusion of highly selective binding of adenosine remained true for both methods.

Another important parameter was the binding stoichiometry (*N*), which indicated how many adenosine/AMP molecules might interact with the aptamer. According to Table 1, the one-site aptamer binds to adenosine and AMP with a N at 0.8 ± 0.1 and 1.3 ± 0.3, respectively, indicating a 1 : 1 binding model. After cutting one phosphodiester bond, the Res-A10-Right-cut also bound just one adenosine (*N* = 0.9 ± 0.2), but it changed to 1.8 ± 0.3 after excising an adenine from the aptamer backbone. The *N* obtained from ITC verified our expectations that each A10-excised aptamer was able to accept two adenosine molecules, with one situated in the loop and the other in the backbone pocket.

Moreover, Δ*H* and Δ*S* also provide us some information about the driving force for aptamer binding. In the one-site aptamer, a big difference in binding adenosine and AMP was observed. Δ*H* and Δ*S* for binding adenosine were −13.5 ± 1.9 kcal mol^−1^ and −22.4 cal K^−1^ mol^−1^, respectively, indicating enthalpy–driven interaction (base pairing). However, AMP titration changed them to −4.5 ± 0.8 kcal mol^−1^ and 6.5 cal K^−1^ mol^−1^, respectively. The decreased Δ*H* and increased Δ*S* revealed entropy-driven binding forces (*e.g.* base stacking). Since AMP was negatively charged and tended to be repelled by the DNA backbone, base-stacking mainly contributed to binding. Interestingly, when only cutting one phosphodiester bond at the backbone, the Δ*H* and Δ*S* values of the Res-A10-Right-cut binding adenosine jumped to −1.1 ± 0.3 kcal mol^−1^ and 13.5 cal K^−1^ mol^−1^, which were very different from the one-site aptamer. After removing a whole A, Δ*H* and Δ*S* of the A10-excised aptamer binding adenosine were −1.0 ± 0.3 kcal mol^−1^ and 18.7 cal K^−1^ mol^−1^, respectively. Its adenosine binding was mainly driven by base stacking.

### Investigating binding site cooperativity

**Fig. 5:**
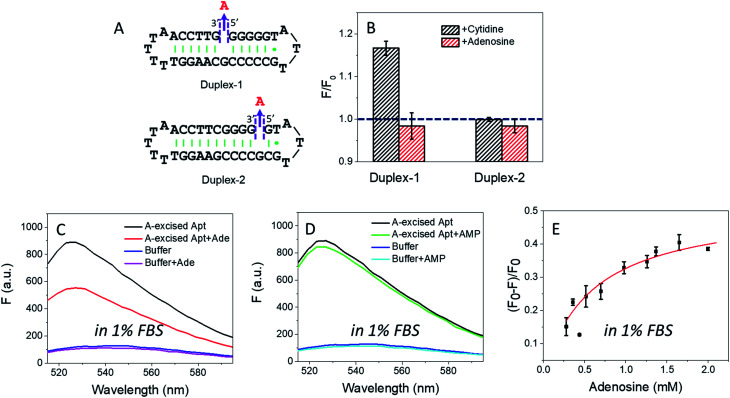
(A) The secondary structure of the designed Duplex-1 and Duplex-2. (B) The fluorescence change of 50 nM Duplex-1 and Duplex-2 with 2 mM ligands (adenosine or cytidine) in buffer A. The Duplex-1 and Duplex-2 were stained by 12.5 nM SGI. Fluorescence spectra of the 50 nM A10-excised aptamer mixed with 2 mM (C) adenosine and (D) AMP in 1% FBS (diluted in buffer A). The DNA was stained by 50 nM SGI. (E) Titrating adenosine (0.28 to 2 mM) with the 50 nM A10-excised aptamer in 1% FBS.

In the wild-type adenosine aptamer, one site can be removed (*e.g.* the one-site aptamer used in this work) and it even slightly improved adenosine binding.^21^ In our A10-excised aptamer, we wanted to understand the relationship between the two adenosine binding sites. For this purpose, two more sequences were designed, named Duplex-1 and Duplex-2, respectively (Fig. 5A). The Duplex-2 exhibited part of the same binding pockets (on the backbone) as the A10-excised aptamer, but its loop pocket was removed and replaced by complementary base pairs (Fig. S2†). If Duplex-2 can recognize the target independently, the free adenosine should be able to fit into the vacancy. Since the right-side of the Duplex-2 is very short, Duplex-1 was also designed with the binding site in the middle to ensure the formation of the flanked hairpins (confirmed by the MFold website server).^53^ If adenosine can be independently recognized in Duplex-2, it should also be recognized in Duplex-1. Therefore, we tested the Duplex-1 first.

Fig. 5B shows that cytidine induced a slight fluorescence increase for Duplex-1 based on the G·C base pairing and stacking (the left black column), but the fluorescence barely changed with adenosine (Fig. 5B, the left red column). Therefore, the two sites in the A10-excised aptamer very likely worked cooperatively, and removing the one from the loop disrupted the binding ability of the other one on the backbone.

For Duplex-2, no fluorescence change was observed regardless of adding cytidine or adenosine. Therefore, this small loop near the 5′-end of the sequence cannot form without the aptamer binding pocket. For comparison, in our A10-excised aptamer, since it showed obvious and strong binding toward adenosine, the aptamer needed to experience a conformational change in its right part.

### Applying the A10-excised aptamer in diluted serum

Based on the data in Fig. 2D, we calculated the detection limit of the A10-excised aptamer to be 46.7 μM adenosine (fitted in Fig. S3†). To demonstrate potential analytical application in a real sample, we then applied the A10-excised aptamer in 1% fetal bovine serum (FBS). Fig. 5C showed that the aptamer retained adenosine binding, with ∼38% fluorescence decrease. AMP still showed no signal (Fig. 5D). The fluorescence signal change in the diluted FBS was close to that in the clean buffers (∼40%, in Fig. 2B). Furthermore, we also titrated adenosine with the A10-excised aptamer in 1% FBS, and calculated *K*_d_ (Fig. 5E). The value was about 0.57 mM, comparable with 0.37 mM in the clean buffer (based on the SGI bind assay). The experiments demonstrated that our A-excised aptamer was robust. The increased background fluorescence disallowed us to test more concentrated serum. Since many signal transduction methods are available based on the wild-type aptamer to ultra-sensitively detect adenosine, the goal of this work is to achieve highly specific adenosine recognition.

## Conclusions

Recently, non-SELEX-derived aptamers were reported based on some classic DNA structures, such as a duplex and a G-quadruplex, through introducing a vacant-site on their backbones. Free nucleosides and nucleotides can re-fit into this vacancy. However, the selectivity of this strategy is still limited likely due to its dependence on these simple secondary structures and interactions (base pairing or even pseudo-base pairing). In this work, we employed an aptamer as a scaffold, which exhibited a more delicate higher structure than a duplex and a G-quadruplex, allowing highly specific adenosine recognition. When excising an entire A from the adenosine aptamer backbone (termed the A-excised aptamer), only adenosine can fit into the vacancy. Other analogues including AMP, ATP, guanosine, cytidine, uridine and theophylline all failed to bind. SYBR Green I (SGI) binding assays and isothermal titration calorimetry (ITC) were used to verify the adenosine binding and investigate related mechanisms. The A10-excised aptamer associated adenosine with a *K*_d_ of 14.8 ± 2.1 μM at 10 °C, mainly driven by base stacking. We demonstrated that the A10-excised aptamer can accept two adenosine molecules, with one in the excised site (DNA backbone) and the other in the original binding pocket (loop area). They worked cooperatively to achieve the high specificity. Finally, this engineered aptamer can also work in diluted FBS. This work provides an intriguing example of using existing aptamer sequences for enhanced functions.

## Conflicts of interest

There are no conflicts to declare.

## Supplementary Material

SC-011-D0SC00086H-s001
