# Supplementary material for: Engineering base-excised aptamers for highly specific recognition of adenosine
Source: Chem Sci. 2020 Feb 10;11(10):2735–43. doi: 10.1039/d0sc00086h (PMC8157715; doi:10.1039/d0sc00086h)
Supplement: SC-011-D0SC00086H-s001 [file SC-011-D0SC00086H-s001.pdf]

Electronic Supplementary Material (ESI) for Chemical Science.
This journal is © The Royal Society of Chemistry 2020

Electronic Supplementary Information

Engineering Base-Excised Aptamer for Highly Specific Recognition of Adenosine

Yuqing Li, Biwu Liu, Zhicheng Huang, Juewen Liu*

Department of Chemistry, Waterloo Institute for Nanotechnology

University of Waterloo

Waterloo, Ontario N2L 3G1, Canada

Phone: (+1) 519 888 4567 ext. 38919

Email: liujw@uwaterloo.ca

Table S1. The DNA sequences used in this work.

**DNA Names**	**Sequences (from 5′ to 3′) and modifications**
Wide-type adenosine Apt	ACCTGGGGGAGTATTGCGGAGGAAGGT
One-site Apt	ACCTTCGGGGAGTATTGCGGAGGAAGGT
A10-excised Apt	GTATTGCGGAGGAAGGTTTTTAACCTTCGGGG
Res-A10-Right cut	GTATTGCGGAGGAAGGTTTTTAACCTTCGGGGA
Res-A10-Left cut	AGTATTGCGGAGGAAGGTTTTTAACCTTCGGGG
Duplex-1	GGGGGTATTGCCCCCGCAAGGTTTTTAACCTTG
Duplex-2	GTATTGCGCCCCGAAGGTTTTTAACCTTCGGGG

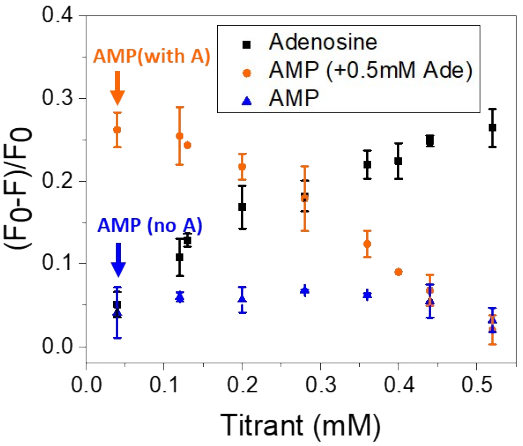

Figure S1. Titrating Adenosine or AMP (0.04-0.52 mM) into 50 nM A10-excised Apt in buffer A (20 mM HEPES, 100 mM NaCl, 5 mM $MgCl_2$). In orange curve, the DNA was first incubated with 0.5 mM adenosine, then applied to AMP titrations. 50 nM SGI was used to indicate the adenosine or AMP binding to DNA.

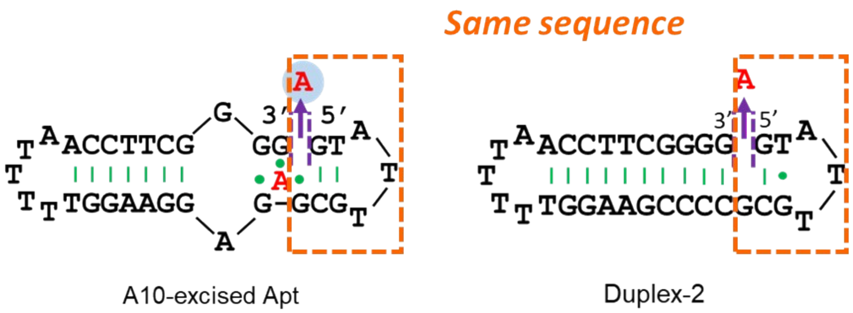

Figure S2. The secondary structure of A10-excised Apt and Duplex-2. The regions in the yellow boxes had the same sequence.

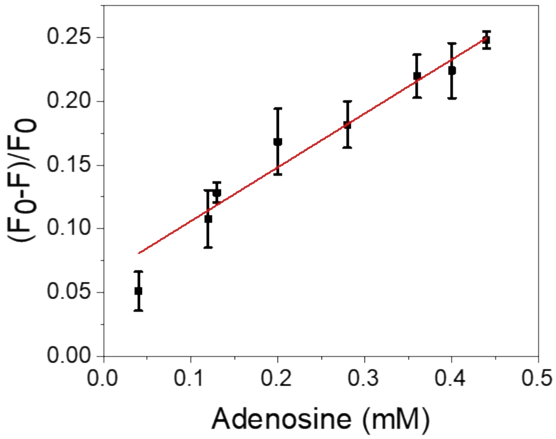

Figure S3. Linear fitting data for titrating adenosine (0.04-0.44 mM) into 50 nM A10-excised Apt in buffer A (20 mM HEPES, 100 mM NaCl, 5 mM $MgCl_2$). 50 nM SGI was used to screen the adenosine binding. The correlation equation is $(F_0\text{-}F)/F_0 = 0.42[\text{Adenosine}] + 0.06$. Based on the equation of 3σ/k, the limit of detection was calculated to be 46.7 μM.
